# 95 Incidence of Hypertrophic Scar Diagnosis in Burn Patients Prescribed Glucocorticoids

**DOI:** 10.1093/jbcr/irac012.097

**Published:** 2022-03-23

**Authors:** Alejandro A Joglar, George Golovko, Shivan N Chokshi, Tsola A Efejuku, Kendall Wermine, Sunny Gotewal, Giovanna De La Tejera, Elvia L Villarreal, Kassandra K Corona, Phillip H Keys, Juquan Song, Steven E Wolf, Amina El Ayadi

**Affiliations:** University of Texas Medical Branch, Galveston, Texas; University of Texas Medical Branch at Galveston, Galveston, Texas; University of Texas Medical Branch at Galveston, Galveston, Texas; University of Texas Medical Branch, Galveston, Texas; University of Texas Medical Branch, Galveston, Texas; University of Texas Medical Branch, Galveston, Texas; University of Texas Medical Branch, Galveston, Texas; University of Texas Medical Branch, Galveston, Texas; University of Texas Medical Branch, Galveston, Texas; University of Texas Medical Branch, Galveston, Texas; University of Texas Medical Branch, Galveston, Texas; University of Texas Medical Branch at Galveston, Galveston, Texas; University of Texas Medical Branch at Galveston, Galveston, Texas

## Abstract

**Introduction:**

Despite advancements in burn care, the optimal treatment to prevent or treat hypertrophic scars is still elusive. Therefore, the objective of this study is to compare the efficacy of five glucocorticoid medications commonly used in the treatment of hypertrophic scarring in burned patients using a large patient database.

**Methods:**

Patients diagnosed with hypertrophic scarring, hypertrophic disorders of the skin, or scar conditions and fibrosis of skin at least one day after burn injury were identified in the TriNetX database. Hydrocortisone, methylprednisolone, dexamethasone, triamcinolone, and prednisone were the glucocorticoids investigated. Those who received a glucocorticoid on the same day or any time after the incidence of burn injury were compared to those who did not take glucocorticoids in the previous five years. Patients were stratified into four groups based on percent total body surface area (TBSA) burned: 0-9%, 10-19%, 20-39%, and 40-100%. A total of 165,041 burned patients were found who did not receive glucocorticoids, and 66,652 burn patients who received glucocorticoids after injury. Statistical analysis for comparison included a risk ratio with a significance defined as a p-value < 0.05.

**Results:**

In all burn patients identified, the risk of hypertrophic scarring diagnosis was reduced with methylprednisolone (RR=0.60, p< 0.001) and prednisone (RR=0.37, p< 0.001), while it was increased with dexamethasone (RR=2.48, p< 0.001). Stratification based on %TBSA burned showed that diagnosis of hypertrophic scarring was reduced in the < 10% TBSA group with methylprednisolone (RR=0.49, p< 0.001) and prednisone (RR=0.33, p< 0.001), while it was increased with dexamethasone (RR=3.6, p< 0.001). Similarly, in the 10-19% TBSA group, the risk was reduced with prednisone (RR=0.57, p=0.024) while increased with dexamethasone (RR=2.2, p< 0.001). No significant effect was observed with hydrocortisone or triamcinolone with any of the %TBSA groups examined. Patients treated with dexamethasone continued to show increased risk for hypertrophic scar diagnosis with 20-39% TBSA (RR=1.69, p< 0.001) and 40-100% TBSA (RR=1.87, p< 0.001).

**Conclusions:**

While methylprednisolone and prednisone decreased the risk of hypertrophic scarring diagnosis among all burn patients identified, dexamethasone showed an increased risk of hypertrophic scarring diagnosis in all burn patients and in each %TBSA stratified group.

